# Assessment of Differences in Clinical Activity and Medicare Payments Among Female and Male Radiation Oncologists

**DOI:** 10.1001/jamanetworkopen.2019.0932

**Published:** 2019-03-22

**Authors:** Luca Valle, Julius Weng, Reshma Jagsi, Fang-I Chu, Sumayya Ahmad, Michael Steinberg, Ann Raldow

**Affiliations:** 1 Department of Radiation Oncology, University of California, Los Angeles; 2 David Geffen School of Medicine, Los Angeles, California; 3 Department of Radiation Oncology, University of Michigan, Ann Arbor; 4 New York Eye and Ear Infirmary of Mount Sinai, New York

## Abstract

**Importance:**

Although physician sex is known to influence salary even after controlling for productivity, sex-based differences in clinical activity and reimbursement among radiation oncologists are poorly understood.

**Objectives:**

To evaluate differences by sex in productivity, breadth of practice, and payments and to characterize Medicare reimbursement by sex among similarly productive groups of radiation oncologists.

**Design, Setting, and Participants:**

A retrospective cohort study was conducted using the January 1 to December 31, 2016, Centers for Medicare & Medicaid Services Physician and Other Supplier Public Use File (POSPUF) to identify charge and payment information for individual radiation oncologists. Clinicians were part of a population-based sample of US radiation oncologists who bill Medicare in both non–facility-based (NFB) and facility-based (FB) practice settings. Analysis was conducted from June 5 to 25, 2018.

**Main Outcomes and Measures:**

Outcome measurements included physician productivity (measured by number of Medicare charges), physician payments (reported as total Medicare payments as well as mean payments per charge submitted and per beneficiary treated), and physician breadth of practice (measured by number of unique Medicare billing codes) in NFB and FB settings.

**Results:**

A total of 4393 radiation oncologists (1133 women and 3260 men) were included in the POSPUF in 2016. Compared with their male counterparts, female physicians in the NFB setting submitted a mean of 1051 fewer charges (95% CI, –1458 to –644; *P* < .001), collected a mean of $143 610 less in revenue (95% CI, –$185 528 to –$101 692; *P* < .001), and used a mean of 1.32 fewer unique billing codes (95% CI, –2.23 to –0.41; *P* = .004). Compared with their male counterparts, female radiation oncologists in the FB setting submitted a mean of 423 fewer charges (95% CI, –506 to –341; *P* < .001), collected a mean of $26 735 less in revenue (95% CI, –$31 910 to –$21 560; *P* < .001), and submitted a mean of 1.28 fewer unique billing codes (95% CI, –1.77 to –0.78; *P* < .001). Women represented 46 of the 397 most highly productive radiation oncologists in the FB setting (11.6%) and collected a mean of $33 026 less (95% CI, –$52 379 to –$13 673; *P* = .001) than men who were similarly productive. In the NFB setting, women represented 54 of the 326 most highly productive radiation oncologists (16.6%) and collected $345 944 (95% CI, –$522 663 to –$169 225; *P* < .001) less than similarly highly productive men. Women collected a mean of $8.49 less per charge (95% CI, –$14.13 to –$2.86; *P* = .003) than men in the NFB setting.

**Conclusions and Relevance:**

This study suggests that female radiation oncologists submit fewer Medicare charges, are reimbursed less per charge they submit, and receive lower Medicare payments overall compared with male radiation oncologists. Even among similarly productive radiation oncologists, women in this study still collected less revenue than men. Further research is required to understand the sex-based barriers to economic advancement within radiation oncology.

## Key Points

**Question:**

How do clinical activities and Medicare payments to radiation oncologists vary by sex?

**Findings:**

This population-based cohort study of the Centers for Medicare & Medicaid Services Physician and Other Supplier Public Use File database demonstrated that regardless of practice setting, female radiation oncologists submitted significantly fewer charges, billed using significantly fewer unique codes, and collected significantly less Medicare reimbursement than did male radiation oncologists; in the non–facility-based setting, women also collected significantly less per charge submitted and per beneficiary treated. Among similarly productive radiation oncologists, women still collected significantly less Medicare reimbursement than did men.

**Meaning:**

The findings suggest that female sex is associated with decreased Medicare reimbursement, and further research is required to understand the mechanisms and potential sex-based barriers to economic advancement within radiation oncology.

## Teaser

This population-based cohort study uses the Centers for Medicare & Medicaid Services Physician and Other Supplier Public Use File to evaluate sex differences among radiation oncologists in productivity, breadth of practice, and payments and to characterize Medicare reimbursement by sex among similar groups of radiation oncologists.

## Introduction

Substantial salary gaps between male and female physicians have long been documented.^1,2,3^ Numerous studies have described the trend of men earning more than women at many stages of their careers,^3^ but these differences are often attributed to different career choices made by men and women in medicine, including the decision to enter less remunerative specialties and work fewer hours. However, among a homogeneous group of academic physicians, a substantial unexplained salary gap has been shown to persist even after adjustment for specialty, academic productivity, and work hours.^4^

Within the field of radiation oncology, sex has been associated with many aspects of a physician’s career, including opportunities for academic advancement,^5^ scholarly activity,^6^ funding for physician researchers,^5^ academic position,^7^ and disease site(s) treated.^8^ Much less is known about differences in payments and clinical activity among male and female radiation oncologists.

To aid in increasing financial transparency in the US health care system, the Center for Medicare & Medicaid Services mandated the publishing of individual physician reimbursements from Medicare via the Physician and Other Supplier Public Use File (POSPUF). This has enabled the objective characterization of differential productivity and reimbursement patterns among male and female radiation oncologists in both the non–facility-based (NFB) and facility-based (FB) practice settings.

Given the interest in better understanding and addressing sex inequities within the field of radiation oncology,^9^ we sought to describe contemporary differences in payments among male and female radiation oncologists submitting claims to Medicare in 2016. We aimed to determine sex-based differences in (1) productivity (measured by number of Medicare charges), (2) payments (reported as total Medicare collections as well as mean and median collections per charge submitted), and (3) breadth of practice (measured by number of unique Medicare billing codes) in the NFB and FB settings. We also sought to provide an overview of sex differences in Medicare payments among similarly productive groups of radiation oncologists.

## Methods

### Physician Payment Database

We queried the Center for Medicare & Medicaid Services’s POSPUF from June 5 to 25, 2018, to identify charge and payment information for radiation oncologists at the individual physician level for the year 2016. Published in 2014, this database links Healthcare Common Procedure Coding System (HCPCS) codes to the National Provider Identifier of each physician submitting charges to Medicare.^10^ Payment (ie, collection) was defined as Medicare reimbursement for a given clinician. As all data were publicly available, this study qualified for the University of California, Los Angeles Institutional Review Board exemption and did not require informed consent. This study followed the Strengthening the Reporting of Observational Studies in Epidemiology (STROBE) reporting guideline.

For each National Provider Identifier, the number of services, the mean charges a physician submitted (total charges divided by number of services), and the mean Medicare payment (total payments divided by number of services) are all reported in POSPUF for a given HCPCS code and practice setting (FB vs NFB). To preserve patient privacy, the Center for Medicare & Medicaid Services does not include any line item performed for 10 or fewer Medicare beneficiaries. Demographic information about each physician, including name, sex, credentials, and practice location are also associated with each National Provider Identifier. The database does not include reimbursement information from other payers, demographic information about beneficiaries treated, or other information about clinicians, including disease sites treated or years in practice.

In both the FB and NFB settings, to determine the Medicare reimbursement for a given clinician, the mean Medicare payment amount for each HCPCS code was multiplied by the number of services provided for that code and then summed to obtain the total collection for that clinician. The number of charges for each clinician was obtained by summing the number of services provided for each clinician. The number of unique HCPCS codes each physician submitted between January 1 and December 31, 2016, was also computed. Means and medians of charges, collections, and unique billing codes for female and male radiation oncologists were tabulated.

Consistent with other reports,^11^ physician productivity in this data set was defined according to the number of unique Medicare claims submitted. To evaluate the influence of clinical productivity on collections, all radiation oncologists were categorized by the number of charges submitted to Medicare. Charge cutoffs used to define productivity groups were based on the 12.5%, 25%, 37.5%, 50%, 62.5%, 75%, and 87.5% quantile for number of charges submitted. Mean and median collections by sex were then calculated for each group.

Our analysis was separated by practice setting, as reimbursements and productivity vary based on the context where physicians operate and bill. The NFB setting includes freestanding outpatient clinics, schools, assisted living facilities, and federally qualifying health centers. In contrast, FB settings are hospital based.

### Statistical Analysis

Normality assumption was imposed and verified by quantile-quantile plot. The *F* test for equal variance of interested variables between the sexes was first conducted. On the result of respective *F* testing, 2-sided 2-sample *t* tests with equal variance or without equal variance were performed to assess the difference of the interested variable between the sexes for all listed comparisons of payment. The Wilcoxon rank-sum test was also used to compare the medians of payment between the sexes. The median differences and 95% CIs were estimated via quantile regression models. Two-sided 2-sample proportion test was conducted to assess the difference in proportion. For all statistical tests, *P* < .05 was considered significant. All analyses were carried out in R, version 3.3.2 (R Foundation for Statistical Computing).^12^

## Results

### POSPUF Physician Demographics

A total of 4393 radiation oncologists (1133 women and 3260 men) were included in the 2016 POSPUF (eTable 1 in the Supplement). Some physicians practiced and billed in both settings; 847 of the 3172 physicians (26.7%) billing in the FB setting were women, whereas 615 of the 2608 physicians (23.6%) billing in the NFB setting were women.

### Sex Differences in Payments, Number of Charges, and Unique Billing Codes

In the NFB setting, a total of 2068 radiation oncologists billed Medicare. A total of 8 755 308 charges representing 447 unique billing codes were submitted by radiation oncologists, who collected $1 060 273 137 in total Medicare payments (Table 1; eFigure 1 in the Supplement). When examining collections, charges, and unique billing codes in the NFB setting, men made 7 184 451 of the charges (82.1%) and collected $877 740 016, or 82.8% of the Medicare reimbursement to radiation oncologists. Female physicians practicing in the NFB setting submitted a mean of 1051 fewer charges (95% CI, –1458 to –644; *P* < .001) and a median of 619 fewer charges (95% CI, –827 to –372; *P* < .001) than their male counterparts. They also collected a mean of $143 610 less in revenue (95% CI, –$185 528 to –$101 692; *P* < .001) and a median of $74 853 less in revenue (95% CI, –$108 194 to –$43 095; *P* < .001). When examining breadth of practice in the NFB setting, female physicians used a mean of 1.32 fewer billing codes (95% CI, –2.23 to –0.41; *P* = .004) and a median of 2.0 fewer billing codes (95% CI, –2.0 to 0; *P* = .002) than their male counterparts.

**Table 1.** Payments, Number of Charges, and Unique Billing Codes in 2016 in Non–Facility-Based Setting

Variable	Total, No. (%)	Mean (SD)	Mean Difference (95% CI)	*P* Value (*t* Statistic)	Median (IQR)	Estimated Median Difference (95% CI)	Median *P* Value (*z* Score)
Payments, $							
All	1 060 273 137	406 546 (572 095)	NA	NA	163 183 (27 058 to 620 950)	NA	NA
Women	182 533 120 (17.2)	296 802 (407 400)	−143 610 (−185 528 to −101 692)	<.001 (−6.72)	114 011 (23 749 to 426 939)	−74 853 (−108 194 to −43 095)	<.001 (−4.39)
Men	877 740 016 (82.8)	440 411 (610 176)	NA	NA	188 864 (27 871 to 673 851)	NA	NA
Charges, No.							
All	8 755 308	3357 (5506)	NA	NA	1553 (318 to 4123)	NA	NA
Women	1 570 858 (17.9)	2554 (3979)	−1051 (−1458 to −644)	<.001 (−5.06)	1110 (260 to 2939)	−619 (−827 to −372)	<.001 (−4.03)
Men	7 184 451 (82.1)	3605 (5876)	NA	NA	1729 (346 to 4582)	NA	NA
Unique billing codes, No.							
All	447	14.48 (10.06)	NA	NA	14 (6 to 21)	NA	NA
Women	240 (53.7)	13.47 (9.61)	−1.32 (−2.23 to −0.41)	.004 (−2.85)	13 (5 to 19)	−2.0 (−2.0 to 0)	.002 (−2.84)
Men	417 (93.3)	14.79 (10.18)	NA	NA	15 (6 to 21)	NA	NA

Abbreviations: IQR, interquartile range: NA, not applicable.

In the FB setting, 3172 radiation oncologists billed Medicare. A total of 4 452 437 charges representing 342 unique billing codes were submitted by radiation oncologists, who collected $287 749 830 in total Medicare payments (Table 2; eFigure 2 in the Supplement). Men submitted 3 526 346 of the charges (79.2%) and collected $227 511 624, or 79.1% of the Medicare reimbursement. Female physicians submitted a mean of 423 fewer charges (95% CI, –506 to –341; *P* < .001) and a median of 347 fewer charges (95% CI, –444 to –230; *P* < .001) than their male counterparts. Female physicians collected a mean of $26 735 less in revenue (95% CI, –$31 910 to –$21 560; *P* < .001) and a median of $22 302 less in revenue (95% CI, –$29 663 to –$14 090; *P* < .001) than their male counterparts. Finally, male breadth of coding was significantly greater than female breadth of coding, with women submitting a mean of 1.28 fewer unique codes (95% CI, –1.77 to –0.78; *P* < .001) and a median of 2.0 fewer unique codes (95% CI, –2.0 to –1.0; *P* < .001) than men.

**Table 2.** Payments, Number of Charges, and Unique Billing Codes in 2016 in Facility-Based Settings

Variable	Total, No. (%)	Mean (SD)	Mean Difference (95% CI)	*P* Value (*t* Statistic)	Median (IQR)	Estimated Median Difference (95% CI)	Median *P* Value (*z* Score)
Payments, $							
All	287 749 830	90 716 (78 820)	NA	NA	74 689 (29 543 to 130 182)	NA	NA
Women	60 238 207 (20.9)	71 119 (57 610)	−26 735 (−31 910 to −21 560)	<.001 (−10.13)	59 199 (26 122 to 102 252)	−22 302 (−29 663 to −14 090)	<.001 (−7.02)
Men	227 511 624 (79.1)	97 854 (84 129)	NA	NA	81 501 (31 508 to 141 865)	NA	NA
Charges, No.							
All	4 452 437	1404 (1274)	NA	NA	1124 (440 to 2002)	NA	NA
Women	926 091 (20.8)	1093 (899)	−423 (−506 to −341)	<.001 (−10.10)	900 (398 to 1540)	−347 (−444 to −230)	<.001 (−6.83)
Men	3 526 346 (79.2)	1517 (1368)	NA	NA	1247 (464 to 2193)	NA	NA
Unique billing codes, No.							
All	342	12.91 (6.84)	NA	NA	14 (9 to 17)	NA	NA
Women	218 (63.7)	11.97 (6.00)	−1.28 (−1.77 to −0.78)	<.001 (−5.04)	12 (8 to 16)	−2.0 (−2.0 to −1.0)	<.001 (−5.51)
Men	314 (91.8)	13.25 (7.09)	NA	NA	14 (9 to 18)	NA	NA

Abbreviations: IQR, interquartile range; NA, not applicable.

Taken together, in both the NFB and FB-based settings, a total of 13 207 746 charges representing 607 unique billing codes were submitted by radiation oncologists, who collected $1 348 022 967 in total Medicare payments (eTable 2 and eFigure 3 in the Supplement). Male physicians charged and collected significantly more than their female counterparts in 2016: in total, men submitted 10 710 797 charges (81.1%), while women submitted 2 496 949 charges (18.9%). Concomitantly, men also collected $ 1 105 251 640, or 82.0% of all Medicare reimbursement, while women collected $242 771 327, or 18.0%. At the individual physician level, women collected a mean of $124 761 less in revenue (95% CI, –$149 996 to –$99 527; *P* < .001) less than men and a median of $52 949 (95% CI, –$62 710 to –$44 643, *P* < .001) less than men. Of the 607 unique billing codes submitted by radiation oncologists in 2016, men used a broader array of unique billing codes (567 [93.4%]) compared with women (338 [55.7%]; *P* < .001). At the individual physician level, women submitted a mean of 1.96 fewer billing codes (95% CI, –2.47 to –1.46; *P* < .001) and a median of 2.0 fewer billing codes (95% CI, –2.0 to –2.0; *P* < .001) than their male counterparts.

When examining collections per charge and collections per beneficiary in each setting, we found that in the NFB setting, women collected $115.34 per charge, while men collected $123.83 per charge. This translated to women collecting $8.49 less per charge (95% CI, –$14.13 to –$2.86; *P* = .003) than men. Mean collections per charge in the FB setting did not differ significantly between sexes, with women collecting $67.98 and men collecting $69.29 (difference, –$1.31; 95% CI, –$3.09 to $0.46; *P* = .15). When considering both settings in aggregate, with mean collection per charge for women of $89.82 and for men of $96.19, women collected $6.37 less per charge (95% CI, –$9.57 to –$3.17; *P* < .001). Mean collections per beneficiary were also significantly higher in men regardless of practice setting (Figure).

**Figure.**
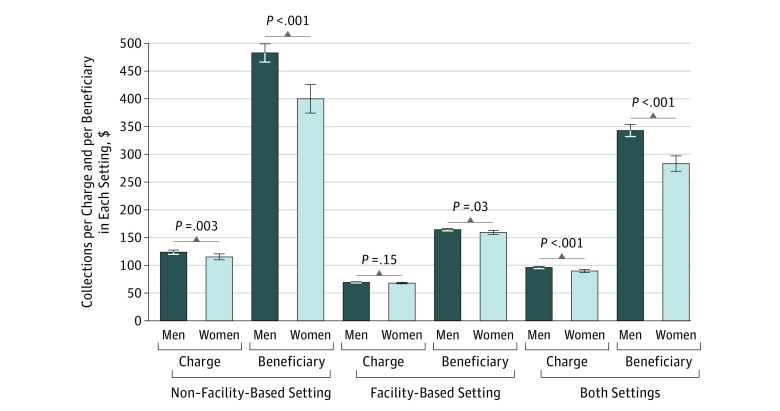
Collections per Charge and per Beneficiary for Men and Women in Each Practice Setting Comparison of mean collections per charge and mean collections per beneficiary by both men and women in the non–facility-based setting, the facility-based setting, and both settings combined. Men consistently collect more dollars per charge as well as per beneficiary. Error bars indicate 95% CIs.

### Physician Sex and Productivity by Number of Charges Submitted

We also examined the association between physician sex and productivity, using the number of charges submitted to Medicare as a proxy for productivity.^11^ In the NFB setting, productivity groups were defined by incremental 12.5% quantiles based on the number of Medicare charges submitted. Women encompassed 25.6% (84 of 328) of the lowest productivity group, but just 16.6% (54 of 326) of the highest productivity group (Table 3). Women in the highest productivity group collected a mean of $345 944 (95% CI, –$522 663 to –$169 225; *P* < .001) less than the men in this highly productive group. The highest productivity group was the only group in which the mean collection difference and the median collection difference between the sexes (median, $121 133; 95% CI, –$356 731 to $8086; *P* = .004) were statistically significant.

**Table 3.** Physician Sex and Productivity by Number of Charges Submitted in 2016 in Non–Facility-Based Setting

Variable	Total, No. (%)	Payments, Mean (SD), $	Mean Difference (95% CI), $	*P* Value (*t* Statistic)	Median Payments (IQR), $	Estimated Median Difference (95% CI), $	Median *P* Value (*z* Score)
Group 1 (cutoff of 87 charges)							
All	328	3703 (3425)	NA	NA	2679 (1143 to 5365)	NA	NA
Women	84 (25.6)	3982 (3142)	375 (−477 to 1228)	.39 (0.87)	3248 (1357 to 5920)	735 (−71 to 2039)	.17 (−0.95)
Men	244 (74.4)	3607 (3518)	NA	NA	2432 (1126 to 5183)	NA	NA
Group 2 (cutoff of 317.75 charges)							
All	324	19 461 (14 145)	NA	NA	15 672 (10 789 to 24 966)	NA	NA
Women	85 (26.2)	19 265 (12 996)	−265 (−3785 to 3254)	.88 (−0.15)	15 639 (10 905 to 26 578)	−346 (−2710 to 2195)	.96 (1.79)
Men	239 (73.8)	19 530 (14 557)	NA	NA	15 985 (10 507 to 24 366)	NA	NA
Group 3 (cutoff of 826.625 charges)							
All	326	56 813 (41 189)	NA	NA	45 781 (30 572 to 69 750)	NA	NA
Women	90 (27.6)	54 524 (36 359)	−3162 (−13 211 to 6886)	.54 (−0.62)	45 214 (30 191 to 61 436)	−271 (−7929 to 7667)	.76 (0.71)
Men	236 (72.4)	57 686 (42 928)	NA	NA	45 906 (30 679 to 72 182)	NA	NA
Group 4 (cutoff of 1553 charges)							
All	326	144 063 (87 443)	NA	NA	134 776 (78 752 to 195 112)	NA	NA
Women	92 (28.2)	136 660 (82 091)	−10 314 (−31 486 to 10 858)	.34 (−0.96)	125 819 (70 090 to 183 753)	−9341 (−42 540 to 28 427)	.19 (−0.89)
Men	234 (71.8)	146 974 (89 461)	NA	NA	135 387 (80 367 to 196 732)	NA	NA
Group 5 (cutoff of 2515.5 charges)							
All	326	260 836 (136 166)	NA	NA	261 882 (140 954 to 340 229)	NA	NA
Women	85 (26.1)	253 334 (123 760)	−10 149 (−43 976 to 23 679)	.56 (−0.59)	256 739 (129 783 to 348 602)	−5276 (−95 127 to 57 299)	.74 (0.65)
Men	241 (73.9)	263 482 (140 428)	NA	NA	262 015 (146 764 to 335 051)	NA	NA
Group 6 (cutoff of 4122.75 charges)							
All	326	483 600 (199 911)	NA	NA	497 903 (361 666 to 603 591)	NA	NA
Women	67 (20.6)	473 534 (189 764)	−12 670 (−66 641 to 41 301)	.64 (−0.46)	496 025 (380 874 to 573 675)	−6898 (−69 564 to 47 957)	.53 (0.08)
Men	259 (79.4)	486 204 (202 726)	NA	NA	502 923 (358 221 to 621 015)	NA	NA
Group 7 (cutoff of 7042.75 charges)							
All	326	837 581 (271 168)	NA	NA	810 835 (685 367 to 979 621)	NA	NA
Women	58 (17.8)	815 542 (238 041)	−26 809 (−104 129 to 50 512)	.50 (−0.68)	825 762 (674 862 to 945 507)	39 399 (−76 092 to 85 685)	.57 (0.17)
Men	268 (82.2)	842 351 (277 994)	NA	NA	810 835 (687 351 to 989 826)	NA	NA
Group 8 (maximum of 81 337 charges)							
All	326	1 446 410 (824 979)	NA	NA	1 317 091 (1 008 834 to 1 708 879)	NA	NA
Women	54 (16.6)	1 157 770 (531 793)	−345 944 (−522 663 to −169 225)	<.001 (−3.88)	1 216 388 (837 567 to 1 475 234)	−121 133 (−356 731 to 8086)	.004 (−2.62)
Men	272 (83.4)	1 503 714 (860 814)	NA	NA	1 354 793 (1 018 617 to 1 780 443)	NA	NA

Abbreviations: IQR, interquartile range; NA, not applicable.

In the FB setting, these trends were preserved (Table 4). Women made up 24.2% (96 of 397) of the lowest productivity group and 11.6% (46 of 397) of the highest productivity group. Women in the highest productivity group collected a mean of $33 026 less (95% CI, –$52 379 to –$13 673; *P* = .001) than men operating with similarly high productivity. Akin to the NFB setting, in the FB setting, the highest productivity group was the only group where mean collection difference and the median collection difference between the sexes (median, –$11 086; 95% CI, –$33 185 to $5757; *P* = .02) were statistically significant.

**Table 4.** Physician Sex and Productivity by Number of Charges Submitted in 2016 in Facility-Based Setting

Variable	Total, No. (%)	Payments, Mean (SD), $	Mean Difference (95% CI), $	*P* Value (*t* Statistic)	Median Payments (IQR), $	Estimated Median Difference (95% CI), $	Median *P* Value (*z* Score)
Group 1 (cutoff of 135.375 charges)							
All	397	4382 (3716)	NA	NA	3344 (1547 to 6469)	NA	NA
Women	96 (24.2)	4068 (3287)	−415 (−1271 to 442)	.34 (−0.95)	3070 (1469 to 6228)	−415 (−1521 to 769)	.51 (0.03)
Men	301 (75.8)	4482 (3843)	NA	NA	3510 (1600 to 6519)	NA	NA
Group 2 (cutoff of 439.75 charges)							
All	396	19 944 (9661)	NA	NA	18 307 (12 880 to 25 305)	NA	NA
Women	133 (33.6)	20 162 (9022)	328 (−1696 to 2351)	.75 (0.32)	19 256 (12 952 to 26 058)	1279 (−1774 to 4156)	.42 (−0.19)
Men	263 (66.4)	19 834 (9984)	NA	NA	17 977 (12 877 to 23 824)	NA	NA
Group 3 (cutoff of 790.125 charges)							
All	397	41 949 (11 869)	NA	NA	40 701 (33 835 to 48 507)	NA	NA
Women	151 (38.0)	41 758 (11 669)	−307 (−2722 to 2108)	.80 (−0.25)	41 330 (33 880 to 48 243)	741 (−2253 to 3682)	.86 (1.07)
Men	246 (62.0)	42 066 (12 013)	NA	NA	40 553 (33 857 to 48 545)	NA	NA
Group 4 (cutoff of 1123.50 charges)							
All	396	64 759 (15 274)	NA	NA	64 551 (55 111 to 74 402)	NA	NA
Women	122 (30.8)	64 000 (16 186)	−1096 (−4367 to 2175)	.51 (−0.66)	64 073 (54 455 to 75 372)	−574 (−5260 to 3171)	.85 (1.06)
Men	274 (69.2)	65 096 (14 868)	NA	NA	64 802 (55 138 to 73 351)	NA	NA
Group 5 (cutoff of 1508.75 charges)							
All	396	90 191 (17 864)	NA	NA	88 390 (78 249 to 101 317)	NA	NA
Women	127 (32.1)	88 830 (14 601)	−2004 (−5440 to 1432)	.25 (−1.15)	87 798 (79 162 to 98 875)	−1150 (−5653 to 2180)	.43 (−0.17)
Men	269 (67.9)	90 834 (19 205)	NA	NA	88 947 (77 626 to 102 664)	NA	NA
Group 6 (cutoff of 2002 charges)							
All	398	114 594 (23 126)	NA	NA	113 672 (100 406 to 126 793)	NA	NA
Women	101 (25.4)	114 727 (22 077)	179 (−5065 to 5422)	.95 (0.07)	113 972 (102 964 to 128 028)	749 (−3562 to 6595)	.60 (0.26)
Men	297 (74.6)	114 548 (23 508)	NA	NA	113 223 (100 335 to 125 088)	NA	NA
Group 7 (cutoff of 2740.625 charges)							
All	395	151 211 (27 130)	NA	NA	151 090 (135 651 to 167 831)	NA	NA
Women	71 (18.0)	148 550 (28 867)	−3244 (−10 235 to 3747)	.36 (−0.91)	148 258 (133 177 to 165 522)	−3749 (−11 806 to 6346)	.47 (−0.09)
Men	324 (82.0)	151 794 (26 746)	NA	NA	152 017 (135 883 to 168 068)	NA	NA
Group 8 (maximum of 12 602 charges)							
All	397	238 695 (77 229)	NA	NA	224 967 (196 568 to 266 133)	NA	NA
Women	46 (11.6)	209 496 (59 308)	−33 026 (−52 379 to −13 673)	.001 (−3.41)	216 330 (184 747 to 245 319)	−11 086 (−33 185 to 5757)	.02 (−2.01)
Men	351 (88.4)	242 522 (78 544)	NA	NA	227 421 (197 880 to 270 568)	NA	NA

Abbreviations: IQR, interquartile range; NA, not applicable.

Taken together, in both the NFB and FB settings, women represented 15.8% (87 of 549) of the most highly productive radiation oncologists, whereas they made up 32.2% (177 of 550) of the least productive radiation oncologists (eTable 3 in the Supplement). Women in the lowest productivity group collected a mean of $3019 more (95% CI, $276-$5763; *P* = .03) than the men in this group. However, in the highest productivity group, women collected a mean of $176 885 less (95% CI, –$298 951 to –$54 817; *P* = .005) than their male counterparts.

## Discussion

In the first comprehensive national study of radiation oncology claims, to our knowledge, we quantify contemporary differences in reimbursement and clinical activity among male and female radiation oncologists. Our findings suggest that female radiation oncologists submit fewer charges and collect less Medicare reimbursement than do male radiation oncologists in all practice settings. In the most productive subset of radiation oncologists, women were less represented and collected less revenue, on average, than similarly highly productive men. The gap in Medicare collections may be driven in part by fewer charges submitted by female radiation oncologists as well as decreased payments per charge and decreased payments per beneficiary treated.

Several studies have examined sex disparities in the field of radiation oncology specifically,^5,13,14,15^ but clinical activity and payment have yet to be studied comprehensively. Our study offers an objective depiction of the Medicare payment landscape for radiation oncologists, which adds to our understanding of sex-based differences in oncologic practice and reimbursement. Observational studies such as ours cannot establish causation for the differential patterns we report, but we can speculate on some of their driving factors.

Multiple factors likely underlie our observation that women submitted fewer charges than men. A flexible work schedule and opportunities for part-time employment have been shown to be attractive options for female radiation oncologists^16^; thus, less clinical activity may be a natural consequence of the value-based labor choices that female practitioners make, particularly within a sex-structured society within which women continue to be expected to shoulder the greater share of domestic responsibilities.^17^ However, increasing data support the notion that both men and women, particularly in younger generations, value balance between work and family,^18^ suggesting that the labor choices that female practitioners make may only partially explain our contemporary practice findings. It is also possible that women prioritize time spent with a given patient vs number of patients seen,^19^ and that extra time spent counseling patients is not reflected in HCPCS code volume. An alternative possibility is that the reduced clinical activity of female practitioners is not associated with the intentional practice choices women make and instead is due to factors outside of their control. Overt discrimination has yet to be eliminated from the profession of medicine,^20,21^ and the surgical literature is replete with instances of harassment and bias on the basis of sex.^22,23^ Thus, in a tertiary referral specialty operating in this context, one cannot dismiss the possibility that female radiation oncologists might receive fewer referrals or be allowed fewer opportunities to care for as many patients as their male counterparts. More important, there is no evidence to suggest that women are less competent and have more limited aspirations for their medical careers than men. On the contrary, research has shown that women often surpass men in productivity metrics (including research publications^24^), albeit in later stages of their career.

Akin to other male-dominated fields,^25^ productivity alone is insufficient to explain the sex differences in compensation reported herein, prompting us to examine other causes for differential reimbursement. Our observation that women bill for less remunerative codes is likely an important factor driving differential collections. It is possible that female radiation oncologists may include in their practice a greater proportion of less well-reimbursed technologies such as 3-dimensional conformal therapies (typically used for treating breast cancer^8^), while foregoing more favorably reimbursed technologies such as intensity modulated radiation therapy^26^ (typically used for treating genitourinary malignant neoplasms). These sex-based distinctions in subspecialization may develop because men are more attuned to the possibility of differences in revenue generation, or because radiation oncology subspecialties that women choose (or are encouraged to choose) involve less revenue-generating “communal” attributes rather than more favorably reimbursed “agentic” attributes.^27^ Given that the gap in mean collections per charge was most pronounced in the NFB setting, it is also possible that, in a private practice setting, men may have more seniority and a greater stake in practice ownership, allowing for potential access to a more highly selected group of patients with malignant neoplasms requiring treatments that reimburse more favorably. Finally, when 2 equivalent treatment options are available, it is possible that female radiation oncologists are more likely to select the more cost-effective option, as women in other specialties have been shown to adhere more closely to clinical guidelines,^28^ practice value-based care,^29^ forego costly interventions,^30^ and engage in shared decision-making with patients more frequently.^19^ Our finding that male radiation oncologists were associated with billing significantly more per beneficiary, absent evidence of parallel improvements in outcomes, underscores the potential relevance of sex-based billing practices in addressing value in oncology.

Within the specialty of radiation oncology, other studies have confirmed a sex-based payment differential^13^ while cautioning against interpreting differences in Medicare collection as further evidence of a pay gap between the sexes. Although it is true that Medicare does not reimburse male and female physicians at different rates, sex differences in the number and mixture of services performed and billed have very real implications for gaps in oncologists’ salaries. Quantifying these differences has allowed us to show that women consistently billed for less remunerative services, which is neither expected nor rational. In comparable studies examining similarly productive subspecialists in other fields such as orthopedic surgery, sex-based differences in Medicare collections were not apparent,^31^ so further study is required to determine the factors unique to radiation oncology that result in women submitting more poorly remunerated procedure codes at the same time that men aggregate more favorably remunerated codes.

As practice setting has also been shown to heavily dictate Medicare collections,^13^ our stratified analysis adds perspective that is lacking in other reports and showed that in this study, female physicians still charged and collected less regardless of whether or not they practiced in settings in which billing for technical services was allowed. Although we did not formally compare charge and payment differences between the NFB and FB settings, the uniform increase in the volume of charges and payment in the NFB setting is consistent with the fact that reimbursement for technical fees typically returns directly to physicians.

### Limitations

An important limitation to our study is that Medicare represents just one of many payment streams to physicians; thus, we paint an incomplete picture of total clinically derived income. However, prior attempts to describe payment patterns in radiation oncology have largely been survey based^32^ and subject to inherent subjectivity and selection bias, whereas our study benefits from objectivity and scale. It is unclear if clinical activity or payment patterns differ with other payers such as commercial insurance or Medicare Advantage, but the Medicare population captures a demographic that is highly relevant in radiation oncology practices; thus, we think that useful payment patterns can still be gleaned from this cohort. Our observational data are also limited to a single snapshot in 2016, and examining these same trends at multiple time points and across multiple generations would be useful to add more context to our findings, particularly because other studies have shown that the gap in clinical activity and payment widens with time.^33^ The role of overcoding or undercoding among the sexes was also not addressed in this study, and could be illuminating. As is the case with any single-payer database, limitations in the POSPUF preclude adjusting for potential confounders, including differences between male and female radiation oncologists in non-Medicare payer mix, disease sites treated, years in practice, and distributions of patients with dual eligibility for Medicare and Medicaid. Although informative, analysis of these variables would not add substantially to our conclusion that sex parity in reimbursement has yet to be achieved in radiation oncology, given both the magnitude of the difference we report as well as our finding that highly productive female radiation oncologists still earn less than similarly highly productive male radiation oncologists.

## Conclusions

Understanding how sex is associated with physician activity and reimbursement in oncology is critical to moving toward a more equitable profession, whereas failure to identify and address potential barriers to economic advancement within radiation oncology may collectively weaken the specialty by further discouraging women from entering the field. Although the number of female medical school graduates has been steadily climbing,^34^ there has been a decline in the proportion of female trainees entering the field of radiation oncology during the last decade^35^ and much-needed attention has been focused on why this may be the case. Efforts to increase sex parity in multiple domains of the specialty are ongoing,^36^ and our report advances our understanding of the nature and scope of the disparity.

Our study illustrates a gap between the sexes in Medicare charges and collections for radiation oncologists, the latter of which is possibly attributable to female physicians consistently submitting fewer charges and charging for services that are less well reimbursed. The source of this variation is unknown but warrants further study, as it may have implications for addressing value as well as sex-based barriers for economic advancement within the specialty of radiation oncology and beyond.

## Notes

Supplement. **eTable 1.** Sex Demographics of Radiation Oncologists in Each Setting **eTable 2.** Payments, Number of Charges, and Unique Billing Codes in 2016 in Non–Facility-Based and Facility-Based Settings **eTable 3.** Physician Sex and Productivity by Number of Charges Submitted in 2016 in Non–Facility-Based and Facility-Based Settings **eFigure 1.** Non–Facility-Based Medicare Collection Distribution for Male and Female Radiation Oncologists **eFigure 2.** Facility-Based Medicare Collection Distribution for Male and Female Radiation Oncologists **eFigure 3.** Facility-Based and Non–Facility-Based Medicare Collection Distribution for Male and Female Radiation Oncologists
