# Supplementary material for: Assessment of Differences in Clinical Activity and Medicare Payments Among Female and Male Radiation Oncologists
Source: JAMA Netw Open. 2019 Mar 22;2(3):e190932. doi: 10.1001/jamanetworkopen.2019.0932 (PMC6583310; doi:10.1001/jamanetworkopen.2019.0932)
Supplement: Supplement. — eTable 1. Sex Demographics of Radiation Oncologists in Each Setting eTable 2. Payments, Number of Charges, and Unique Billing Codes in 2016 in Non–Facility-Based and Facility-Based Settings eTable 3. Physician Sex and Productivity by Number of Charges Submitted in 2016 in Non–Facility-Based and Facility-Based Settings eFigure 1. Non–Facility-Based Medicare Collection Distribution for Male and Female Radiation Oncologists eFigure 2. Facility-Based Medicare Collection Distribution for Male and Female Radiation Oncologists eFigure 3. Facility-Based and Non–Facility-Based Medicare Collection Distribution for Male and Female Radiation Oncologists [file jamanetwopen-2-e190932-s001.pdf]

Supplementary Online Content

Valle L, Weng J, Jagsi R, et al. Assessment of differences in clinical activity and Medicare payments among female and male radiation oncologists. *JAMA Netw Open.* 2019;2(3):e190932. doi:10.1001/jamanetworkopen.2019.0932

eTable 1. Sex Demographics of Radiation Oncologists in Each Setting
eTable 2. Payments, Number of Charges, and Unique Billing Codes in 2016 in Non–Facility-Based and Facility-Based Settings
eTable 3. Physician Sex and Productivity by Number of Charges Submitted in 2016 in Non–Facility-Based and Facility-Based Settings
eFigure 1. Non–Facility-Based Medicare Collection Distribution for Male and Female Radiation Oncologists
eFigure 2. Facility-Based Medicare Collection Distribution for Male and Female Radiation Oncologists
eFigure 3. Facility-Based and Non–Facility-Based Medicare Collection Distribution for Male and Female Radiation Oncologists

This supplementary material has been provided by the authors to give readers additional information about their work.

© 2019 Valle L et al. *JAMA Network Open.*

eTable 1. Sex Demographics of Radiation Oncologists in Each Setting

	All Settings	Non Facility-Based (NFB) Setting	Facility-Based (FB) Setting
Total	4,393	2,608	3,172
# of female (%)	1,133 (25.8)	615 (23.6)	847 (26.7)
# of male (%)	3,260 (74.2)	1,993 (76.4)	2,325 (73.3)

Note: some radiation oncologists work in both NFB and FB settings

© 2019 Valle L et al. *JAMA Network Open.*

eTable 2. Payments, Number of Charges, and Unique Billing Codes in 2016 in Non–Facility-Based and Facility-Based Settings

Variable	Total (%)	Mean (SD)	Mean Difference (95% CI)	Mean P Value (t Statistic)	Median (first quartile, third quartile)	Estimated Median Difference (95% CI)	Median P Value (Z score)
**Collections, $**							
All	1,348,022,967	306,857 (463,119)			138,983 (68,611, 313,991)		
Women	242,771,327 (18.0)	214,273 (317,441)	-124,761 (-149,996, -99,527)	<0.001 (-9.69)	100,707 (52,004, 198,808)	-52,949 (-62,710, -44,643)	<0.001 (-10.56)
Men	1,105,251,640 (82.0)	339,034 (500,027)			153,729 (77,713, 364,355)		
**Charges**							
All	13,207,746	3,007 (4,364)			1865 (967, 3,412)		
Women	2,496,949 (18.9)	2,204 (3,028)	-1,082 (-1,321, -842)	<0.001 (-8.86)	1,407 (747, 2,457)	-666 (-800, -548)	<0.001 (-10.90)
Men	10,710,797 (81.1)	3,286 (4,709)			2,073 (1,068, 3,690)		
**Unique Billing Codes**							
All	607	16.56 (8.02)			16 (12,20)		
Women	338 (55.7)	15.10 (7.23)	-1.96 (-2.47, -1.46)	<0.001 (-7.60)	15 (11, 18)	-2 (-2,-2)	<0.001 (-8.88)
Men	567 (93.4)	17.06 (8.22)			17 (13, 21)		

© 2019 Valle L et al. *JAMA Network Open.*

eTable 3. Physician Sex and Productivity by Number of Charges Submitted in 2016 in Non–Facility-Based and Facility-Based Settings

Variable	Total (%)	Mean (SD) Collections, $	Mean Difference (95% CI), $	Mean P Value (t Statistic)	Median Collections (first quartile, third quartile), $	Estimated Median Difference (95% CI), $	Median P Value (z Statistic)
Group 1, With a Cutoff of 468 Charges							
All	550	17,398 (16,499)			13,622 (5,321, 25,522)		
Women	177 (32.2)	19,446 (14,165)	3,019 (276, 5,763)	0.03 (2.16)	16,431 (8,817, 27,293)	3,686 (929, 9,105)	<0.001 (-3.39)
Men	373 (67.8)	16,426 (17,432)			12,745 (4,150, 232,834)		
Group 2, With a Cutoff of 967 Charges							
All	549	58,202 (29,015)			52,263 (41,120, 66,336)		
Women	199 (36.2)	54,951 (22,102)	-5,100 (-9,670, -530)	0.03 (-2.19)	49,925 (42,239, 62,998)	-2,676 (-6,382, 1,247)	0.23 (-0.72)
Men	350 (63.8)	60,051 (32,176)			52,610 (40,761, 68,540)		
Group 3, With a Cutoff of 1,402 Charges							
All	549	101,545 (62,161)			83,220 (72,182, 103,898)		
Women	189 (34.4)	98,488 (52,405)	-4,662 (-14,854, 5,529)	0.37 (-0.90)	83,883 (73,482, 99,766)	707 (-4,244, 4,974)	0.90 (1.26)
Men	360 (65.6)	103,150 (66,728)			83,161 (70,826, 106,019)		
Group 4, With a Cutoff of 1,865 Charges							
All	549	134,529 (62,279)			113,532 (99,022, 140,516)		
Women	153 (27.9)	128,568 (59,589)	-8,264 (-19,899, 3,371)	0.16 (-1.40)	109,427 (95,329, 129,783)	-5,563 (-9,720, 840)	0.04 (-1.75)
Men	396 (72.1)	136,832, (63,210)			115,066 (99,748, 144,341)		
Group 5, With a Cutoff of 2,445 Charges							
All	549	181,293 (96,420)			148,258 (129,339, 184,660)		
Women	129 (23.5)	171,025 (76,676)	-13,423 (-29,910, 3,065)	0.11 (-1.60)	144,193 (126,202, 172,063)	-5,442 (-12,934, 221)	0.09 (-1.34)
Men	420 (76.5)	184,447 (101,591)			149,723 (130,292, 187,268)		
Group 6, With a Cutoff of 3,412 Charges							
All	549	276,340 (157,624)			205,735 (173,747, 376,546)		
Women	117 (21.3)	294,708 (174,622)	23,343 (-8,896, 55,582)	0.16 (1.42)	209,160 (170,532, 426,898)	3,654 (-12,417, 87,292)	0.38 (-0.29)
Men	432 (78.7)	271,365 (152,533)			204,980 (173,990, 344,957)		
Group 7, With a Cutoff of 5,415 Charges							
All	549	484,300 (249,416)			464,733 (254,151, 682,496)		
Women	82 (14.9)	511,730 (243,984)	32,246	0.28 (1.08)	506,213 (249,812, 710,424)	71,901 (20,345, 213,053)	0.37 (-0.34)

© 2019 Valle L et al. *JAMA Network Open.*

			(-26,407, 90,899)				
Men	467 (85.1)	479,484 (250,304)			431,909 (255,086, 680,730)		
Group 8, With a Maximum of 81,337 Charges							
All	549	1,201,777 (738,329)			1,076,707 (811,054, 1462726)		
Women	87 (15.8)	1,052,923 (468,787)	-176,885 (-298,951, -54,817)	0.005 (-2.86)	1,033,761 (763,421, 13,182,95)	-65,143 (-194,783, 45,995)	0.10 (-1.27)
Men	462 (84.2)	1,229,808 (775,913)			1,099,142 (820,798, 1,514,736)		

© 2019 Valle L et al. *JAMA Network Open.*

eFigure 1. Non–Facility-Based Medicare Collection Distribution for Male and Female Radiation Oncologists

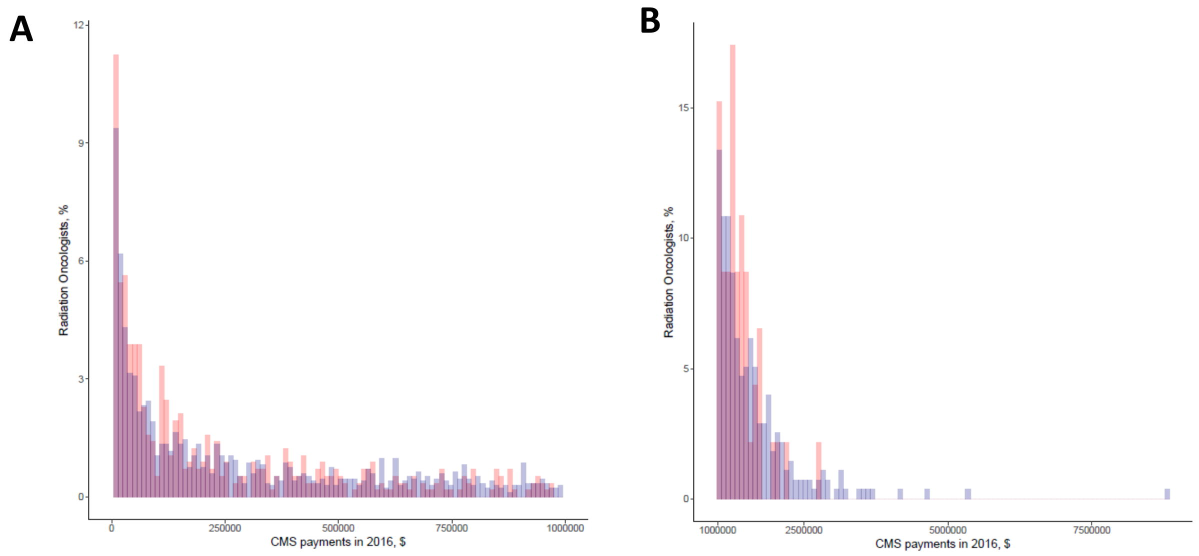

A. Collections equal or less than $1,000,000 in non–facility-based settings. B. Collections exceeding $1,000,000 in non–facility-based settings. Male radiation oncologists are represented in blue and female radiation oncologists in pink

© 2019 Valle L et al. *JAMA Network Open.*

eFigure 2. Facility-Based Medicare Collection Distribution for Male and Female Radiation Oncologists

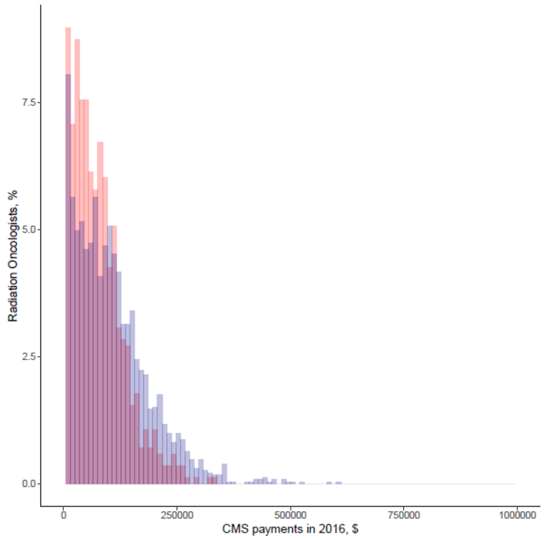

Collections equal or less than $1,000,000 in facility-based settings. Male radiation oncologists are represented in blue and female radiation oncologists in pink

© 2019 Valle L et al. *JAMA Network Open.*

eFigure 3. Facility-Based and Non–Facility-Based Medicare Collection Distribution for Male and Female Radiation Oncologists

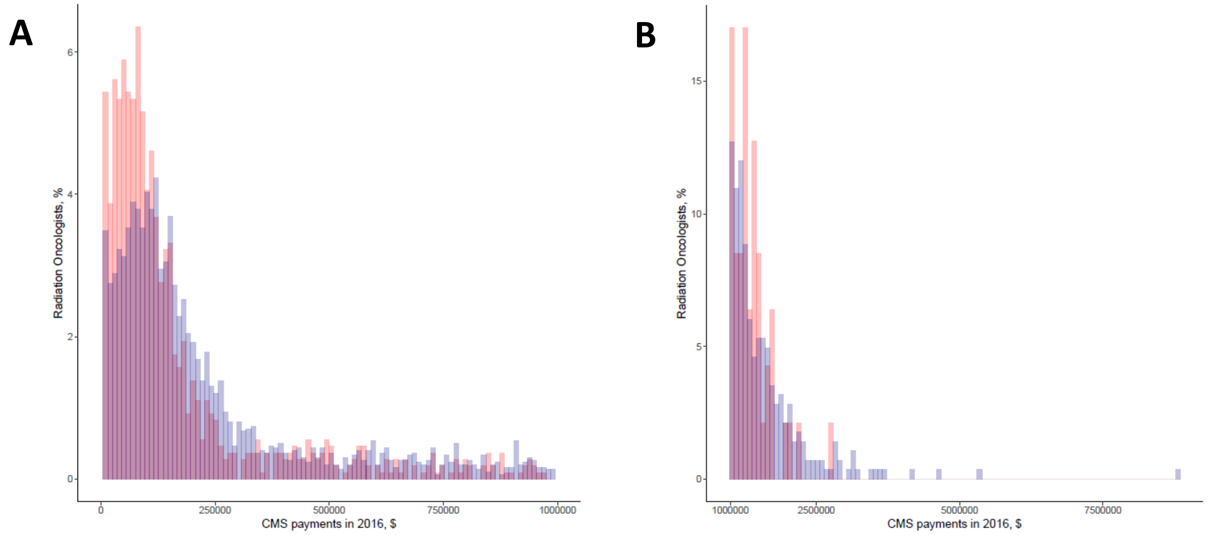

A. Collections equal or less than $1,000,000 in both facility-based and non–facility-based settings. B. Collections exceeding $1,000,000 in both facility-based and non–facility-based settings. Male radiation oncologists are represented in blue and female radiation oncologists in pink

© 2019 Valle L et al. *JAMA Network Open.*
